# Follicular Variant of Papillary Thyroid Carcinoma Presented as Autonomous Functioning Thyroid Nodule: A Case Report and Review of Literature

**DOI:** 10.7759/cureus.3014

**Published:** 2018-07-20

**Authors:** Amir Shahbaz, Mina Fransawy Alkomos, Rupak Mahendhar, Usman Nabi, Maria Riaz, Issac Sachmechi

**Affiliations:** 1 Internal Medicine, Icahn School of Medicine at Mount Sinai/Queen Hospital Center, New York, USA; 2 Research, California Institute of Behavioral Neurosciences & Psychology, Sacramento, USA; 3 Internal Medicine, Icahn School of Medicine, Mount Sinai/Queens Hospital Center, New York, USA; 4 Diagnostic Radiology, Hamad General Hospital, Doha, QAT; 5 Internal Medicine, Icahn School of Medicine at Mount Sinai/Queens Hospital Center, New York, USA

**Keywords:** thyroid nodule, thyroid carcinoma, hyperthyroidism

## Abstract

Follicular variant of papillary thyroid carcinoma (FVPTC) presented as an autonomous functioning thyroid nodule is a rare finding. We reported a case of 70-year-old male presented with complaints of palpitation and heat intolerance. On palpation, we found a thyroid nodule of 4 cm in the left lobe. Thyroid function tests revealed hyperthyroidism, and radioactive iodine uptake scan (RAIU) showed increased uptake in the left lobe consistent with a hot nodule. The probability of the benign nature of hyperfunctioning thyroid nodule discussed but patient requested further workup to rule out any remote possibility of thyroid cancer. We performed a fine needle aspiration (FNA), and the cytological examination suggested the possibility of thyroid carcinoma. The patient underwent total thyroidectomy, and histological examination revealed follicular architecture with nuclear features of papillary carcinoma in 1 cm area of the thyroid nodule. In the review of the literature, we identified the following seven cases of FVPTC arising within a hyperfunctioning thyroid nodule.

## Introduction

A thyroid nodule is a common problem in clinical practice. Thyroid nodule >1 cm in any diameter required a serum thyroid stimulating hormone (TSH) level. If the serum TSH is subnormal, a radioiodine uptake thyroid scan should be obtained to document the functional status of the thyroid nodule. Hyperfunctioning nodules rarely harbor malignancy, and no cytological evaluation is necessary [1]. However, the risk of malignancy may be underestimated in the hot nodule. A recent literature review revealed an estimated 3.1% prevalence [2]. We present a case of a follicular variant of papillary thyroid carcinoma (FVPTC) arising within a hyperfunctioning thyroid nodule.

## Case presentation

**Figure 1:**
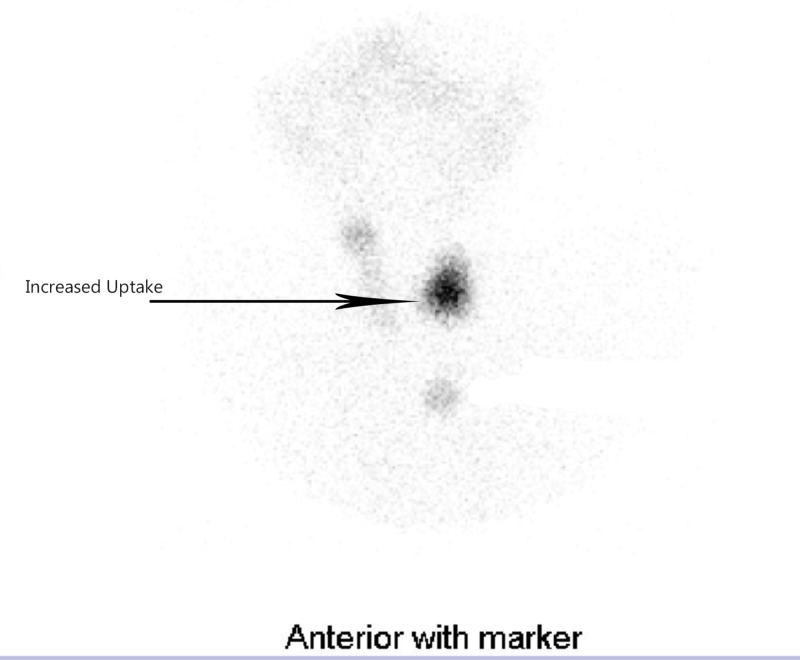
Radioiodine uptake scan. The thyroid scan shows a hot nodule in the left lobe and suppression in the remaining thyroid tissue.

A 70-year-old male, known case of diabetes mellitus, hypertension and coronary artery disease, presented with complaints of increased appetite, weight loss, palpitations and heat intolerance. Physical examination revealed 4 cm thyroid nodule in the left lobe on palpation. His blood pressure was 130/85 mmHg, and resting pulse was 102/min with sinus rhythm. His TSH suppressed 0.29 uIU/mL (Reference range: 0.40–4.00 uIU/mL) while free thyroxine (FT4) 2.1 ng/dL (0.8–1.9 ng/dL) and free triiodothyronine (FT3) 4.2 pg/mL (1.5–4.1 pg/mL) elevated. In Figure 1, the radioiodine uptake scan showed the abnormal focus of hot uptake in the left lobe, suggestive of a hyperfunctioning toxic thyroid nodule.

The patient had classic signs and symptoms of hyperthyroidism. The possibility of the benign nature of hyperfunctioning thyroid nodule discussed, but the patient requested further workup to rule out any remote possibility of thyroid cancer. A fine needle aspiration (FNA) performed, and the cytology report was suggestive of thyroid carcinoma. The patient then underwent total thyroidectomy. The pathology report confirmed the fine needle aspiration cytology (FNAC) finding and revealed a solitary tumor measuring 3.5 cm in diameter. The architecture was predominantly follicular, and papillary cytological features were best seen in an area of 1 cm consistent with a follicular variant of papillary thyroid carcinoma. No other cancerous tissue found in the remaining thyroid gland. Due to the small size of the tumor no ablative radioiodine therapy performed. Post surgery, the patient received levothyroxine to prevent hypothyroidism and to stop TSH stimulation. Serum TSH and serum thyroglobulin were checked regularly. On follow-up visit, radioactive iodine whole-body scan did not reveal any distant metastasis. This case is a rare example of FVPTC arising within a toxic nodule.

## Discussion

The term hot nodule of the thyroid is described as an area of greater radioiodine uptake compared to the surrounding thyroid tissue. The presence of a hot nodule is usually believed to exclude the diagnosis of thyroid cancer [3]. The autonomous functioning thyroid nodule presented as a thyroid cancer requires careful evaluation and consideration. The exact incidence is difficult to quantify; one reason for this is the variability in how hot nodules defined. When malignancy is present it may coexist with hyperfunctioning tissue in the same gland but at different sites [4]. Pazaitou-Panayiotou et al. explored the relationship between thyroid cancer and hyperthyroidism. In seven out of 17 patients with hyperthyroidism associated solitary autonomous adenomas, the tumor located outside the hot nodule [5]. The risk of malignancy in hot nodule increases with family history, large size, rapid growth, irregular outline and lack of mobility within the surrounding tissue [3].

In our case, the patient presented with hyperthyroidism, and localized uptake of ^131^I was consistent with a hot nodule. Fine needle aspiration has a higher false negative rate as the size of the nodule increases. As such, the American Thyroid Association recommends patients with nodules greater than 4 cm who have indeterminate cytology should undergo total thyroidectomy as the first line of treatment [6]. Follicular variant of papillary thyroid carcinoma is found in 9–22.5% of patients with papillary thyroid carcinoma. It characterizes as follicular architecture with nuclear features of papillary carcinoma. The nuclear features are subtle; histological interpretation is prone to intra-observer variation and, as such, mistaken for follicular adenoma [7]. In the review of the literature, we found the following cases of the follicular variant of thyroid carcinoma arising within a hot nodule (Table 1).

**Table 1:** List of reported cases of follicular variant of papillary thyroid carcinoma arising within a hot nodule. RAIU: Radioactive iodine uptake; US neck: Ultrasound neck; FNAC: Fine needle aspiration cytology; PET/CT: Positron emission tomography/computed tomography; FVPTC: Follicular variant of papillary thyroid carcinoma. Reported year = the year case report published

Author	Reported year	Patient age/sex	Presentation	Nodule detected	Treatment	Outcome
Azevedo & Casulari [7]	2010	47/F	Weight loss, nervousness, tremors, fatigue and insomnia	US neck + RAIU + FNAC	Total thyroidectomy + Radioiodine Ablation	Was well on follow visit with no recurrence.
Bommireddipalli et al. [8]	2010	63/M	Five months history of the progressively enlarging neck mass, weight loss, and fatigue	US Neck +RAIU+ FNAC	Total thyroidectomy + Radioiodine Ablation	One year follow-up PET/CT scan revealed a metabolically active pre-tracheal lymph node, which on biopsy was confirmed to be stage III FVPTC.
Ruggeri et al. [9]	2013	15/ F	Symptoms of hyperthyroidism+ had a positive family history of thyroid cancer	US neck + RAIU + FNAC	Total thyroidectomy + Radioiodine Ablation	Not mentioned
Gabalec et al. [10]	2014	15 /F	Hyperfunctioning thyroid nodule	RAIU+ FNAC	Total thyroidectomy+ Radioiodine Ablation	Disease-free on follow-up
Kuan & Tan [11]	2014	60/F	Enlarged thyroid mass, dysphagia, hoarseness, heat intolerance, palpitation and weight loss	US neck + RAIU + FNAC	Total thyroidectomy + Radioiodine Ablation	Not mentioned
Rees et al. [4]	2015	16/F	Thyroid mass, weight loss, tremors, frequent bowel movements and hair loss	US neck+ RAIU+ FNAC	Total thyroidectomy + Radioiodine Ablation	Currently well and under active follow-up
Lima et al. [12]	2018	49/F	Cervical mass, unexplained weight loss, anxiousness, sweating, and insomnia	US neck+ RAIU+ FNAC	Total thyroidectomy + Radioiodine Ablation	Asymptomatic at follow-up

The presence of a follicular variant of papillary carcinoma of thyroid associated with hyperfunctioning thyroid nodule is rare. It is clinically very important that the finding of a hot nodule may indicate thyroid malignancy. After total thyroidectomy due to the potential for metastatic spread, a radioactive iodine whole-body scan should be performed to detect for residual disease. Patients require lifelong thyroid hormone replacement therapy and measurement of serum TSH and serum thyroglobulin for follow-up. This case highlights the importance of careful evaluation of a thyroid nodule to determine whether it is malignant or not.

## Conclusions

The presence of a hot nodule cannot always rule out thyroid cancer. Therefore, careful evaluation of hot nodule is necessary so that malignancy does not get overlooked.
